# Effects of adjunctive brexpiprazole on calmness and life engagement in major depressive disorder: post hoc analysis of patient-reported outcomes from clinical trial exit interviews

**DOI:** 10.1186/s41687-021-00380-4

**Published:** 2021-12-11

**Authors:** Catherine Weiss, Stine R. Meehan, T. Michelle Brown, Catherine Gupta, Michael F. Mørup, Michael E. Thase, Roger S. McIntyre, Zahinoor Ismail

**Affiliations:** 1 Otsuka Pharmaceutical Development & Commercialization Inc., 508 Carnegie Center Drive, 1 University Square Drive, Princeton, NJ 08540 USA; 2 H. Lundbeck A/S, Valby, Copenhagen, Denmark; 3 RTI Health Solutions, Research Triangle Park, NC USA; 4 Perelman School of Medicine, University of Pennsylvania and the Philadelphia Veterans Affairs Medical Center, Philadelphia, PA USA; 5 Mood Disorders Psychopharmacology Unit, University Health Network, University of Toronto, Toronto, ON Canada; 6 Departments of Psychiatry, Clinical Neurosciences, and Community Health Sciences, Hotchkiss Brain Institute and O’Brien Institute for Public Health, University of Calgary, Calgary, AB Canada

**Keywords:** Adjunctive, Antidepressant, Brexpiprazole, Calmness, Clinical trial, Engagement, Major depressive disorder, Patient-reported outcome

## Abstract

**Background:**

Though often overlooked, calming patients and increasing their life engagement are key factors in the treatment of major depressive disorder (MDD). This study aimed to test the hypothesis that adjunctive brexpiprazole increases calmness and life engagement among patients with MDD, based on clinical trial exit interviews.

**Methods:**

This was a pooled analysis of exit interview data from three exploratory, open-label studies of adjunctive brexpiprazole 1–3 mg/day. The studies enrolled 105 outpatients with MDD (DSM-IV-TR criteria), a current depressive episode, and inadequate response to antidepressant treatment during the current episode. Patients were interviewed if they completed the end-of-treatment visit (Week 6 or Week 12, depending on the study). Exit interviews took the form of semi-structured telephone interviews in which patients were asked mostly qualitative questions about their symptoms prior to the start of the study, and about improvements they had noted during treatment. Interview transcripts were reviewed and codes were assigned to calmness and life engagement vocabulary, allowing aggregation of the frequency of improvement in various domains.

**Results:**

79.8% (83/104) of patients described improvements consistent with at least one calmness term, most commonly feeling less anxious (46.2%) or less irritable (44.2%). A four-domain concept of patient life engagement was developed in which 88.6% (93/105) of patients described improvements consistent with at least one domain, specifically, emotional (77.1%), physical (75.2%), social (41.9%), and/or cognitive (36.2%). Of the patients who described improvement in calmness, 96.4% (80/83) also described improvement in life engagement.

**Conclusions:**

Analysis of exit interview data suggests that patients were calmer and more engaged with life following treatment with adjunctive brexpiprazole. Thus, adjunctive brexpiprazole may provide a benefit on subjective patient outcomes in addition to the improvement in depressive symptoms shown by clinical rating scale data.

*Trial Registration*: Data used in this post hoc analysis came from ClinicalTrials.gov identifiers: NCT02012218, NCT02013531, NCT02013609.

**Supplementary Information:**

The online version contains supplementary material available at 10.1186/s41687-021-00380-4.

## Background

Major depressive disorder (MDD) is a common mental health disorder characterized by periods of depressed mood and/or loss of interest or pleasure in activities [1]. Clinical trials of potential new drugs for MDD generally assess efficacy using clinician-rated depression symptom scales, such as the Hamilton Depression Rating Scale (HAM-D) or the Montgomery–Åsberg Depression Rating Scale (MADRS) [2, 3]. However, the use of total scores on such scales does not reflect the different endophenotypes of depression that have been identified by factor analysis, such as affective, vegetative, cognitive/retardation and anxiety/agitation [4–9]. Furthermore, clinician-rated scales do not measure a patient’s subjective experience of treatment, and may not reflect the patient’s treatment goals, which often differ from those of the clinician [10, 11]. As a consequence of this discrepancy, approximately half of patients who are in remission based on a clinician-rated scale do not consider themselves to be in remission based on self-reports [10].

Patient ‘life engagement’ is a broad term that describes positive health aspects relating to cognition (including ‘hot’ cognition, i.e., cognition colored by emotion), vitality, motivation and reward, and the ability to feel pleasure. It reflects the functional outcomes of life fulfillment, well-being, and participation in valued and meaningful activities [12]. In one study, patients with MDD ranked such outcomes—specifically ‘life being meaningful’, ‘enjoyment of life’, and ‘satisfaction with oneself’—as the most important indicators of treatment success [11]. Symptoms of a low level of life engagement (i.e., anhedonia, loss of interest, lack of motivation) are key drivers for the prescription of an adjunctive antipsychotic in MDD [13]. At the other end of the spectrum, symptoms of overactivation (i.e., agitation, hostility, irritability) are also key drivers for antipsychotic use in MDD [13]. The term ‘calmness’ describes the mental state of peace, serenity, and tranquility, which includes being free from agitation, excitement, worry, or disturbance. Calming a patient (without sedation) and increasing their life engagement (without overactivation) are key components of the successful treatment of MDD. Despite their importance to patients, such aspects of depression are rarely studied.

Brexpiprazole is a serotonin–dopamine activity modulator that acts as a partial agonist at serotonin 5-HT_1A_ and dopamine D_2_ receptors, and as an antagonist at serotonin 5-HT_2A_ and noradrenaline α_1B_/α_2C_ receptors, all with subnanomolar affinity [14]. In randomized controlled trials in MDD, adjunctive brexpiprazole was found to be efficacious and well tolerated among adults with inadequate response to antidepressant treatments (ADTs) [15–19]. These findings were supported by exploratory open-label studies in several MDD subpopulations [20–22].

Unsolicited feedback to the drug manufacturer from health care professionals and patient call centers suggests that patients with MDD appear to be calmer and more engaged with life after taking adjunctive brexpiprazole. The observation of improved patient life engagement is supported by a post hoc analysis of clinical trial data, in which adjunctive brexpiprazole improved life engagement versus placebo as measured by an exploratory patient-reported outcome measure [23]. Clinical trial exit interviews can be used to complement data from clinical rating scales by documenting the patient perspective and experience with a treatment [24, 25], and are therefore directly relevant to clinical practice. The aim of the present analysis was to test the hypothesis that adjunctive brexpiprazole increases calmness and life engagement among patients with MDD, based on exit interviews from three exploratory studies.

## Methods

### Study design and patients

In this post hoc analysis, exit interview data were pooled from three short-term, exploratory, open-label Phase 3b studies of adjunctive brexpiprazole in patients with MDD (ClinicalTrials.gov identifiers: NCT02012218, NCT02013531, and NCT02013609) [20–22]. The studies were conducted at multiple sites in the United States between November 2013 and October 2014.

Full descriptions of the studies have been published [20–22]. In brief, the studies enrolled male and female outpatients with a Diagnostic and Statistical Manual of Mental Disorders, Fourth Edition, Text Revision (DSM-IV-TR) criteria diagnosis of MDD [26] and a current depressive episode. Study 1, a switching study, enrolled 61 adults (aged 18–65 years) who had an inadequate response to ≥ 1 adjunctive treatments in the current episode. Study 2 enrolled 37 adults (aged 18–65 years) who had anxiety symptoms and an inadequate response to 1–3 ADTs in the current episode. Study 3 enrolled 48 young adults (aged 18–35 years) who were working or in school (at least part-time) and who had an inadequate response to 1–3 ADTs in the current episode.

In each study, after a ≤ 21 day screening phase, patients entered a treatment phase in which oral brexpiprazole 1–3 mg/day was added to a patient’s current ADT for 6 weeks (Studies 1 and 2) or 12 weeks (Study 3). Brexpiprazole was started at 0.5 mg/day and titrated over 2 weeks to a target dose of 2 mg/day; dose increases/decreases within the range of 1–3 mg/day were allowed from the Week 3 visit onwards, based on the investigator’s judgement.

MADRS, Clinical Global Impressions—Severity of illness (CGI-S), and Sheehan Disability Scale (SDS) scores were measured at baseline and at periodic intervals during the studies. The MADRS is a validated, 10-item, clinician-rated measure of depressive symptom severity in MDD, with total scores ranging from 0 (least severe) to 60 (most severe) [27, 28]. The CGI-S is a single-item, clinician-rated measure of overall mental illness severity, scored from 1 (not at all ill) to 7 (among the most extremely ill) [29]. It is widely used in psychiatry, though its validity has been questioned [30]. The SDS is a validated, 3-item, patient-rated measure of functional impairment in psychiatric disorders; SDS Mean score ranges from 0 (least impairment) to 10 (most impairment) [31, 32].

### Exit interviews

All English-speaking patients who completed the end-of-treatment visit (Week 6 in Studies 1 and 2; Week 12 in Study 3) were eligible for an exit interview. Interviews were conducted by telephone in a 10-day window prior to the end-of-treatment visit.

A copy of the exit interview guide is presented in the Additional file 1: Appendix 1. The goal of the exit interviews was to supplement clinical trial data by assessing patients’ overall experiences with MDD before the trial, and their perception of treatment benefits as they completed the trial. The interviews were semi-structured and mostly qualitative in nature. After a brief overview of the interview process, the patient was asked to spontaneously report the symptoms they had prior to the start of the study. Next, the patient was asked to describe symptoms on which they felt they had improved during the treatment phase of the study; if not spontaneously reported, the following target symptoms were prompted: anxiety, irritation/irritability, agitation, impulsivity, aggression, anger/hostility, physical health, energy/motivation.

Each interview was conducted by two employees of RTI Health Solutions: (1) either Dr. Brown or Dr. DiBenedetti, who are experienced and trained interviewers, and (2) a note taker. Each interview was anticipated to take approximately 60 min, and was audio recorded. Anonymized transcripts were prepared in a standardized, quality-controlled manner, with each transcript undergoing multiple levels of review.

### Defining ‘calmness’ and ‘life engagement’

In order to determine the effects of adjunctive brexpiprazole on calmness and life engagement, it was first necessary to define these terms. Stages in the development of a definition of ‘calmness’ were (1) analysis of exit interview transcripts by employees of RTI Health Solutions to identify all reported improvements of any nature; (2) selection of terms related to the concept of calmness from the full list of improvements, by employees of RTI Health Solutions; and (3) review and approval of the final definition by the authors.

Stages in the development of a definition of ‘life engagement’ were (1) a panel discussion with expert psychiatrists and employees of RTI Health Solutions on the concept of life engagement; (2) a review of transcripts and field notes from the exit interviews by employees of RTI Health Solutions to identify domains of life engagement and examples of ‘low’ and ‘high’ life engagement; and (3) review and approval of the final definition by expert psychiatrists.

### Analysis

Data were included for all patients who completed an exit interview, provided they met the eligibility criteria described in the ‘Exit interviews’ section, above.

Codes relating to components of the calmness and life engagement definitions were assigned to the exit interview vocabulary for each patient, allowing aggregation of the frequency of improvement in each domain. Calmness codes were assigned by a process of content analysis, in which a patient’s exact words and phrases were matched to the a priori calmness definition. Life engagement codes were assigned by a process of thematic analysis, in which concepts from the field notes (and the transcripts, where more context was required) were matched to the a priori life engagement definition using an iterative approach, with collaboration between coders where needed to reach consensus on new related concepts or specific cases in context. Duplicates in a single domain were counted once only. More than 10% of symptoms were independently double coded by two different coders, and any discrepancies were resolved by the coders in discussion with Drs. Brown and/or DiBenedetti.

No formal statistical analysis was conducted on the exit interview data. Descriptive statistics are provided, as appropriate, at the aggregate level. Analyses were performed in Excel® for Office 365 (Microsoft; Redmond, WA), and Venn diagrams were plotted using eulerAPE [33].

In addition, least squares mean changes in MADRS Total, CGI-S, and SDS Mean scores were calculated from baseline to Week 6 of adjunctive brexpiprazole treatment, stratified by whether patients described improvement in calmness and/or engagement in the exit interviews. The rating scales were analyzed with a linear regression model, controlling for baseline score. These analyses were performed using R-3.5.1, with the packages haven, tidyverse, reshape2, kableExtra, lme4, emmeans, sas7bdat, readxl, plyr, stringr, dplyr, writexl, and nlme [34].

## Results

### Patients

Of the 112 patients who completed one of the studies, exit interviews were completed and analyzed for 103 patients (92.0%): 50/51 from Study 1, 30/32 from Study 2, and 23/29 from Study 3. Reasons for not completing an exit interview and/or for being excluded from the analysis were: 6 patients could not be reached despite multiple attempts; 1 patient did not speak English; 1 patient reported during the interview having discontinued brexpiprazole; and 1 interview was conducted outside of the permitted 10-day window. Following a discussion with the study sponsors, data were included for an additional 2 patients who completed an exit interview and then discontinued the study prior to the end-of-treatment visit, meaning that a total of 105 exit interviews were analyzed. Due to an audio recording malfunction at the time of the interview, 1 interview had no transcript; the interview notes were sufficient to include the patient in the engagement analysis but not in the calmness analysis.

Pooled baseline demographic data were available for 103 of the 105 interview participants. The mean (standard deviation) age was 42.5 (14.1) years across the total sample: 46.5 (12.4) years in Study 1, 46.2 (14.6) years in Study 2, and 28.2 (5.2) years in Study 3 (the young adult study). In total, 77 participants (74.8%) were female.

### Definitions

Illustrative quotations from the exit interviews are presented in the Additional file 1: Appendix 2.

#### Definition of calmness

From the interview transcripts, 73 distinct codes (patient-reported words or phrases) were identified relating to improvements of any nature (Additional file 1: Table S1 in Appendix 3). Of these codes, 29 were related to calmness and thereby formed the definition of calmness (Table 1A).

**Table 1:** Definitions of (A) calmness and (B) life engagement in major depressive disorder

**A Patient-reported improvement codes related to calmness**		
Aggravated (less)	Frustration (less)	Peaceful (more)
Aggressive (less)	Hostility (less)	Physical tightness/stiffness (less)
Agitated (less)	Impulsivity (less)	Relaxed (more)
Anger (less)	Irritability (less)	Restlessness (less)
Anxiety (less)	Knot in chest/stomach (resolved)	Shakiness (less)
At ease (more)	Mellow (more)	Sit still (improved)
Calm (more)	Nervousness (less)	Stress/stressed over things (less)
Edginess (less)	Overwhelmed (less)	Tension (less)
Fear (less)	Panic/panic attacks (less)	Worry (less)
Fidgety (less)	Patient (more)	

B Four-domain conceptual framework of life engagement		
Domain	Low	High
Emotional (affect/mood)	Negative affect/mood, flat, depressed	Positive affect/mood, happy, hopeful
Physical (energy)	Low energy, fatigued, tired	High energy, energized, motivated, productive
Social (interest)	Low involvement, disengaged, anhedonia, disinterested	Involved, engaged, interested
Cognitive (alertness/thinking)	Low arousal, brain fog, slowed thinking	High arousal, alert, clarity, attentive, aware

#### Definition of life engagement

Following discussions with an expert panel and a review of interview transcripts and field notes, a four-domain conceptual framework of patient life engagement was devised (Table 1B).

### Application of definitions to exit interviews

#### Calmness

**Fig. 1:**
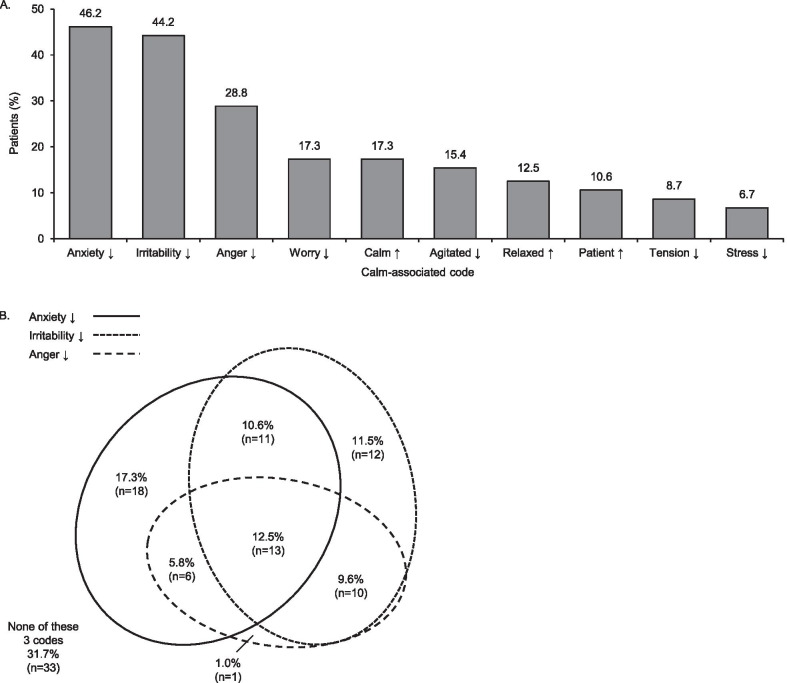
**A** Frequency that specific calmness terms were described as being improved (top ten); and **B** overlap of references to improved anxiety, irritability, and anger (n = 104). ↑ = more; ↓ = less

Of the 104 patients with interview transcripts, 83 (79.8%) described improvements consistent with at least one calmness term. The most frequently referenced calmness terms are presented in Fig. 1A (the full list is given in Additional file 1: Table S1 in Appendix 3). Nearly half of patients reported feeling less anxious or less irritable.

The majority of patients used multiple calmness terms; however, reference to multiple terms will partly reflect a patient’s expressiveness of speech during the interview, as many of the codes are synonyms. The most frequently overlapping calmness terms were reductions in anxiety, irritability, and anger (Fig. 1B).

Five of the calmness codes (agitated, fidgety, restlessness, shakiness, and sit still) could potentially be affected by akathisia. When these five terms were excluded from the analysis, 81/83 patients still described improvements consistent with at least one calmness term.

Patients who described improvement in calmness, versus those who did not describe improvement in calmness, had a greater mean improvement in MADRS Total, CGI-S, and SDS Mean scores during the studies they had just completed (Table 2).

**Table 2:** Mean change from baseline to Week 6 in efficacy rating scale scores

Improvement in calmness	MADRS Total score		CGI-S score		SDS Mean score	
	Yes (n = 83)	No (n = 20)	Yes (n = 83)	No (n = 20)	Yes (n = 83)	No (n = 20)
Mean (SD) at baseline	29.7 (5.3)	29.5 (6.5)	4.3 (0.5)	4.5 (0.5)	6.4 (1.7)	6.8 (1.6)
LS mean (SE) change to Week 6	− 18.5 (0.8)	− 13.9 (1.8)	− 2.1 (0.1)	− 1.4 (0.2)	− 3.4 (0.3)	− 2.1 (0.6)
Difference (95% CLs)	− 4.57 (− 0.81, − 8.34)		− 0.64 (− 0.17, − 1.11)		− 1.36 (− 0.11, − 2.62)	
*p* value	0.018		0.0082		0.033	

Improvement in life engagement	MADRS Total score		CGI-S score		SDS Mean score	
	Yes (n = 92)	No (n = 11)	Yes (n = 92)	No (n = 11)	Yes (n = 92)	No (n = 11)
Mean (SD) at baseline	29.8 (5.4)	29.0 (7.0)	4.3 (0.5)	4.6 (0.5)	6.5 (1.7)	6.4 (1.4)
LS mean (SE) change to Week 6	− 18.7 (0.8)	− 9.3 (2.1)	− 2.1 (0.1)	− 0.8 (0.3)	− 3.5 (0.3)	− 0.9 (0.7)
Difference (95% CLs)	− 9.39 (− 4.95, − 13.83)		− 1.36 (− 0.82, − 1.90)		− 2.58 (− 1.09, − 4.07)	
*p* value	< 0.0001		< 0.0001		0.0009	

Improvement in both calmness and life engagement	MADRS Total score		CGI-S score		SDS Mean score	
	Yes (n = 80)	No (n = 23)	Yes (n = 80)	No (n = 23)	Yes (n = 80)	No (n = 23)
Mean (SD) at baseline	29.7 (5.0)	29.0 (5.8)	4.3 (0.5)	4.5 (0.5)	6.4 (1.7)	6.8 (1.6)
LS mean (SE) change to Week 6	− 18.3 (0.8)	− 13.3 (1.6)	− 2.1 (0.1)	− 1.4 (0.2)	− 3.5 (0.3)	− 1.9 (0.5)
Difference (95% CLs)	− 5.04 (− 1.57, − 8.51)		− 0.75 (− 0.32, − 1.18)		− 1.65 (− 0.49, − 2.81)	
*p* value	0.0048		0.0009		0.0057	

Stratified by whether the patient described improvement in calmness and/or life engagement in their exit interview. Analysis includes 103 patients with a baseline and a Week 6 efficacy measurement

*CGI-S* Clinical Global Impressions–Severity of illness, *CL* confidence limit, *LS* least squares, *MADRS* Montgomery–Åsberg Depression Rating Scale, *SD* standard deviation, *SDS* Sheehan Disability Scale, *SE* standard error

#### Life engagement

The assignment of life engagement codes to exit interview vocabulary is presented at the individual patient level in Additional file 1: Table S2 in Appendix 3.

**Fig. 2:**
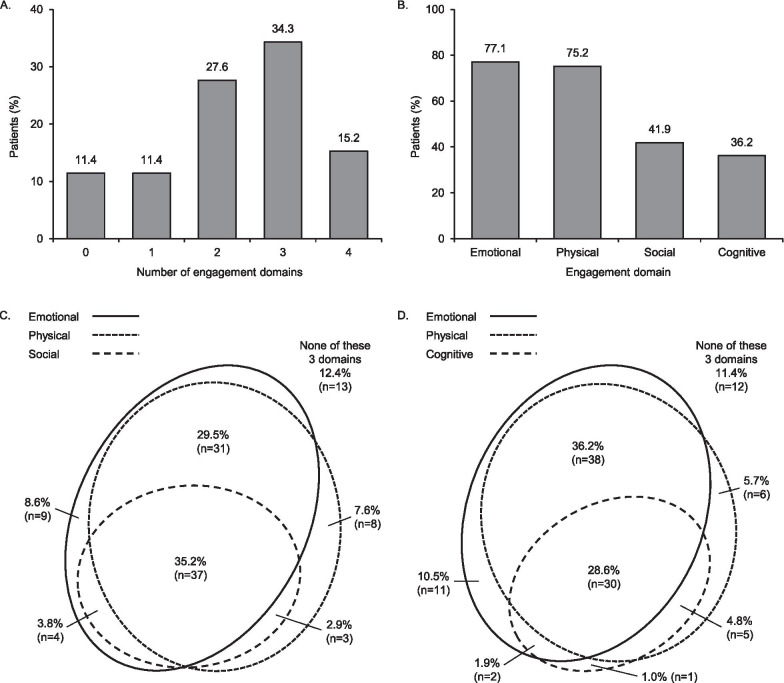
**A** Number of life engagement domains that each patient described as being improved; **B** frequency that specific life engagement domains were described as being improved; and **C**, **D** overlap of references to emotional, physical, and social/cognitive engagement domains (n = 105)

Of the 105 interviewed patients, 93 (88.6%) described improvements consistent with at least one life engagement domain. Most commonly, patients described improvements consistent with two or three domains (Fig. 2a).

Approximately three quarters of patients described improvement consistent with the emotional domain, and similarly for the physical domain. The social and cognitive domains were less frequently referenced (Fig. 2b).

The most frequently overlapping domains (i.e., multiple domains described as improving by the same patient) were the emotional and physical domains (Fig. 2c, d).

Patients who described improvement in life engagement, versus those who did not describe improvement in life engagement, had a greater mean improvement in MADRS Total, CGI-S, and SDS Mean scores during the studies they had just completed (Table 2).

#### Overlap of calmness and life engagement

**Fig. 3:**
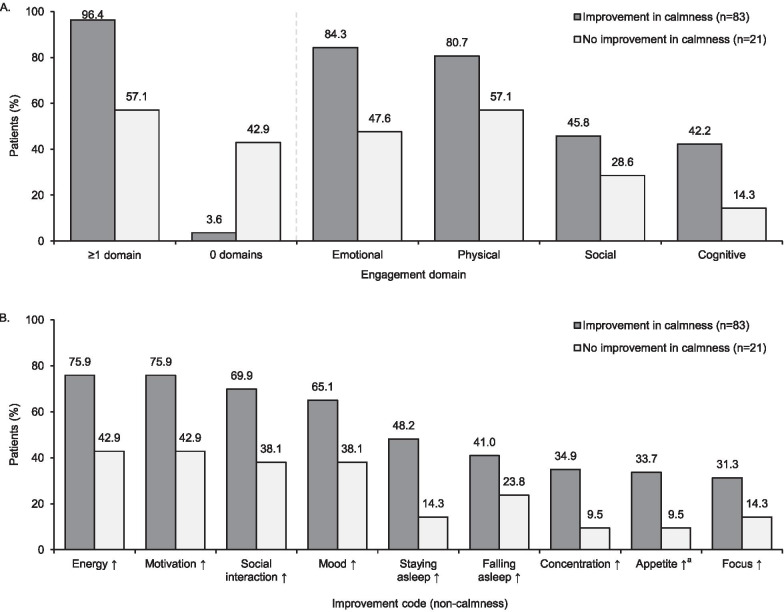
**A** Frequency that life engagement domains were described as being improved, and **B** frequency that specific non-calmness terms were described as being improved (top nine), stratified by whether the patient described improvement in calmness (n = 104). ^a^Improved (less or more, depending on the individual). ↑ = more/improved

Of the 83 patients who described improvement in calmness (at least one calmness term), 80 (96.4%) also described improvement in life engagement (at least one engagement domain). Most commonly, these patients described improvements consistent with the emotional and physical domains (Fig. 3a). Among patients who described improvement in calmness, the most frequently referenced non-calmness terms (from the list of 73 improvements of any nature) were more energy, more motivation, and improved social interactions (Fig. 3b).

Of the 21 patients who did not describe improvement in calmness, 12 (57.1%) described improvement in life engagement (Fig. 3a).

Patients who described improvement in both calmness and life engagement, versus those who described improvement in neither/just one, had a greater mean improvement in MADRS Total, CGI-S, and SDS Mean scores during the studies they had just completed (Table 2).

## Discussion

In this analysis of exit interview data from three open-label studies, patients described being calmer and more engaged with life following treatment with adjunctive brexpiprazole, supporting the hypothesis that adjunctive brexpiprazole increases calmness and life engagement among patients with MDD.

Based on a new definition of calmness comprising 29 terms derived from patient vocabulary, approximately 80% of patients described feeling more calm following adjunctive brexpiprazole treatment. The calmness was largely driven by patients’ reports of feeling less anxious, irritable, and angry, but many individuals also described feeling less worried and agitated, and feeling more calm (i.e., specifically using the word ‘calm’), relaxed, and patient. Thus, whereas certain other atypical antipsychotics can reduce symptoms of anxiety in MDD [35], increased calmness as described by patients receiving adjunctive brexpiprazole encompassed more than just a lack of anxiety.

The term ‘life engagement’ was considered appropriate to describe the benefits of adjunctive brexpiprazole that related to life fulfillment, well-being, and valued living [12]. A four-domain conceptual framework of life engagement was devised based on exit interview transcripts and discussion with expert psychiatrists. This approach follows the trend to conceptualize mental disorders based on dimensions of observable behavior and neurobiological measures, such as those of the National Institute of Mental Health (NIMH) Research Domain Criteria (RDoC) project [36]. The resulting dimensions—emotional, physical, social, and cognitive—encompass terminology that aligns with outcomes considered important to patients with depression [37]. In the present study, nearly 90% of patients receiving adjunctive brexpiprazole described improvement in one or more life engagement domains, most commonly the emotional and physical domains. There was considerable overlap between improvement in calmness and life engagement, showing that the calming effect was not at the detriment of reduced activity in daily life. Conceptually, while there may be overlap between calmness and life engagement, calmness is related to improvement of anxiety, irritability, and dysphoria, whereas life engagement includes improvement of anhedonia and apathy. In particular, the three dimensions of apathy in the 2018 apathy diagnostic criteria (behavior/cognition, emotion, and social interaction) show considerable overlap with the life engagement domains developed in the present study (emotional, physical, social, and cognitive) [38]. Despite the overlapping domains, anhedonia and apathy do not fully capture the concept of life engagement described in Table 1 (lacking, for example, terms relating to attention, alertness, and clarity of thought). Similarly, the concepts of life satisfaction and quality of life, though widely used in psychiatry, do not fully capture the energy and cognitive aspects of life engagement that were described by patients receiving adjunctive brexpiprazole treatment. Having developed this preliminary definition of life engagement, future research needs to validate the concept from the patient perspective, and clarify how it differs from existing concepts.

Patients who described improvements in calmness and/or life engagement, versus those who did not, had a greater improvement on formal rating scales for depressive symptoms, global severity, and functioning during the studies they had just completed. Although limited by the fact that stratification was based on spontaneous reports of improvement, this result suggests that the patient-reported exit interview data align with validated clinical rating scale data. In part, this may be attributed to a degree of overlap between the clinician-rated outcomes and patient reports (e.g., the MADRS ‘inner tension’ item overlaps with calmness terminology, and the ‘concentration difficulty’ item overlaps with the cognitive domain of life engagement). Overall, these data suggest a benefit of adjunctive brexpiprazole on subjective patient outcomes in addition to the improvement in depressive symptoms shown by clinical rating scale data [15].

Calmness and life engagement are desirable effects, which should be distinguished from the antipsychotic side effects of sedation (somnolence, sedation, fatigue) and activation (akathisia, restlessness, agitation, anxiety, insomnia), respectively. Of the other agents approved by the US Food and Drug Administration as either adjunctive treatments for MDD or treatment of treatment-resistant depression, aripiprazole is associated with activating side effects (particularly, akathisia) and sedating side effects, and quetiapine extended release and olanzapine–fluoxetine combination are associated with sedating side effects [39]. Certain activating effects may be linked to high intrinsic activity at, for example, the dopamine D_2_ receptor [14], whereas sedation may be linked to high affinity at, for example, the histamine H_1_ receptor [40, 41]. Brexpiprazole has a lower intrinsic activity than aripiprazole at the D_2_ receptor and moderate affinity for the H_1_ receptor; consequently, brexpiprazole has shown a low rate of activating and sedating side effects in clinical trials [14–19].

The use of exit interviews reflects the increasing recognition of the importance of patient-reported outcomes (PROs) in clinical research [42]. A PRO—the Inventory of Depressive Symptomatology Self-Report (IDS-SR)—was included in the Phase 3 randomized controlled trials of adjunctive brexpiprazole in MDD [43]. In parallel with the present analysis, an expert panel selected relevant items from the IDS-SR that represent patient well-being and life engagement [23]. Using this ‘IDS-SR_10_ Life Engagement’ subscale, adjunctive brexpiprazole showed a benefit on items reflecting hot cognition, vitality, motivation and reward, the ability to feel pleasure, and subjective well-being—meaningful benefits to patients, which reflect the four-domain concept of life engagement used in the present study.

This analysis is limited by its post hoc, open-label design, and by the lack of active adjunctive comparator such as another approved adjunctive antipsychotic. A relatively small number of patients was included, reducing the generalizability of results. Regarding questionnaires, answers may be associated with recall bias, the data were subjectively extracted from transcripts, and the questions specifically addressed ‘improved’ symptoms without consideration to potential worsening of symptoms. The definition of life engagement was developed after the interviews had been completed, meaning that patients were not specifically asked to describe life engagement in their own words, or if the concept of life engagement resonated with them; a future study will address these issues and incorporate patient feedback into the definition. With regard to the calmness analysis, exclusion of five calmness codes that may be affected by akathisia had minimal impact on the patient sample, suggesting that the overall calm-related improvements were not driven by akathisia-related improvements. Evidently, further work is needed to validate the definition of calmness and the concept of patient life engagement in four domains.

## Conclusions

Analysis of exit interview data suggests that patients were calmer and more engaged with life following treatment with adjunctive brexpiprazole. Thus, adjunctive brexpiprazole may provide a benefit on subjective patient outcomes in addition to the improvement in depressive symptoms shown by clinical rating scale data. Increased calmness and life engagement are desirable (though often overlooked) outcomes in MDD, and should be taken into consideration when designing a treatment plan that is effective across all areas of a patient’s life.

## Supplementary Information

**Additional file 1**.** Appendix 1**. A copy of the interview guide.** Appendix 2**. Illustrative quotations from the exit interviews.** Table S1 Appendix 3**. Additional results: Patient-reported improvement codes relating to improvements of any nature (n = 104).** Table S2 in Appendix 3**. Additional results: Assignment of life engagement codes to exit interview vocabulary (n = 105).

## Data Availability

To submit inquiries related to Otsuka clinical research, or to request access to individual participant data (IPD) associated with any Otsuka clinical trial, please visit https://clinical-trials.otsuka.com/. For all approved IPD access requests, Otsuka will share anonymized IPD on a remotely accessible data sharing platform.

## Abbreviations

ADT: Antidepressant treatment
CGI-S: Clinical Global Impressions–Severity of illness
DSM-IV-TR: Diagnostic and Statistical Manual of Mental Disorders, Fourth Edition, Text Revision
HAM-D: Hamilton Depression Rating Scale
IDS-SR: Inventory of Depressive Symptomatology Self-Report
MADRS: Montgomery–Åsberg Depression Rating Scale
MDD: Major depressive disorder
NIMH: National Institute of Mental Health
PRO: Patient-reported outcome
RDoC: Research Domain Criteria
SDS: Sheehan Disability Scale
