# Supplementary material for: Effects of adjunctive brexpiprazole on calmness and life engagement in major depressive disorder: post hoc analysis of patient-reported outcomes from clinical trial exit interviews
Source: J Patient Rep Outcomes. 2021 Dec 11;5:128. doi: 10.1186/s41687-021-00380-4 (PMC8665966; doi:10.1186/s41687-021-00380-4)
Supplement: Supplementary file 1 — Additional file 1. Appendix 1. A copy of the interview guide. Appendix 2. Illustrative quotations from the exit interviews. Table S1 Appendix 3. Additional results: Patient-reported improvement codes relating to improvements of any nature (n = 104). Table S2 in Appendix 3. Additional results: Assignment of life engagement codes to exit interview vocabulary (n = 105). [file 41687_2021_380_MOESM1_ESM.pdf]

Supplementary material

Supplement to: Effects of adjunctive brexpiprazole on calmness and life engagement in major depressive disorder: post hoc *analysis of patient-reported outcomes from clinical trial exit interviews*

Contents

Appendix 1. A copy of the interview guide 2

Appendix 2. Illustrative quotations from the exit interviews 8

Calmness-associated quotations 8

Life engagement-associated quotations 9

Appendix 3. Additional results 10

Supplementary Table 1. Patient-reported improvement codes relating to improvements of any nature (n=104) 10

Supplementary Table 2. Assignment of life engagement codes to exit interview vocabulary (n=105) 12

Appendix 1. A copy of the interview guide

Interview date _______________(DDMMMYYYYY)

Study Patient Study ID# __________

ASSESSING TREATMENT BENEFIT OF BREXPIPRAZOLE: EXIT INTERVIEW GUIDE

I. INTRODUCTION

[Introduce interviewer]

[Confirm respondent's name prior to starting the interview]

[Remind subject of the purpose of the interview (Note: details were already covered in the consent form but are briefly reviewed below)]

- I want to briefly go over some of the information about today's interview, some of which was covered in the study informed consent form.
- The purpose of this interview today is to learn more about your experiences during the clinical study that you just completed at **[Name of clinic/doctor]**. Specifically, our discussion today will focus on your experiences with depression as well as some associated symptoms and behaviors both before and during this clinical study.
- We are also interested in any improvements you may have noticed during the course of the clinical study (if any). The information you provide today will be used to help the study sponsors better understand people's experiences with depression and the impact of treatment.
- As we go along, please feel free to speak openly and share your opinions freely. There are no wrong answers here - you are the expert.
- As a reminder, our discussion today is scheduled to take about 60 minutes. Also, we are audio-recording all interviews to make sure we do not miss any important information and to help us write a summary report. The audio-recordings will be transcribed and both the transcripts and audio-recordings given to the study sponsor. Your name and other identifying information is removed during the transcript process.
- Unless you report a side effect associated with brexpiprazole during today's call, your name will never be associated with anything you share with us.
- If you do report a side effect associated with brexpiprazole during today's call, I have to let your clinic staff know and they will determine if they need to follow up with you for more information.
- Do you have any questions before we begin the interview?

Interview date ________________(DDMMMYYYYY)

Study Patient Study ID# __________

____

II. MDD SYMPTOMS PRIOR TO STUDY

First, I would like to learn a little more about your experiences with depression at the time you started the clinical study.

1. At the start of treatment in this study, how were you feeling and what were you experiencing as related to your depression? What were your main issues? Anything else? **[Obtain exhaustive listing of depression symptoms when the study drug was started]**
    a. When you say **[symptom/issue]**, what exactly does that mean to you? **[Be sure to understand participants' interpretation of each symptom and specific components of any complex concepts such as anxiety, e.g., is it more physical anxiety or mental (worry)?]**

III. CLINICAL STUDY IMPROVEMENTS

Now, I'd like you to think back over the last **[6/12]** weeks of the brexpiprazole study.

2. You have been treated for depression in this study and may, or may not, have experienced some improvements. Tell me how you think you may have improved. **[Have patients describe each change/improvement reported; obtain listing of all improved symptom areas]**

3. **[If not spontaneously reported, probe for understanding of improvement in target symptoms listed below]:**
    a. **Anxiety (i.e., feeling nervous, uneasy, worried, cannot relax, or tense)**
    b. **Irritation or irritability (i.e., feeling impatient, aggravated, and easily annoyed, losing your temper)**
    c. **Agitation (i.e., feeling like you have to move or cannot sit still)**
    d. **Impulsivity (i.e., behaving without thinking first; behaviors that occur quickly without planning or giving it much thought)**
    e. **Aggression (i.e., feeling or acting hostile or angry; acting out)**
    f. **Anger and hostility (probe on these and how they may relate or are similar to the other listed symptoms, feelings, and behaviors**
    g. **Physical health: (i.e., general health, occurrence of aches, pains, stomach or breathing problems)**
    h. **Energy/motivation (i.e., strength and vitality necessary for sustained physical or mental activity)**

4. Based on your experience with the study medication, are some of the improvements more important than others? Which ones? How so? **[List the most improved symptoms]**
   a. What are your 3 most important improvements? **[Obtain the TOP 3 most important improvements]**

5. Before we talk more about your improvements in your depression and related symptoms, I would like to understand how you would rate these symptoms at the start of the clinical study. Using a **scale from 0, meaning "not at all" to 10, meaning "as bad as it can be,"**, how would you rate your level of…
   b. **[MOST IMPORTANT IMPROVED SYMPTOM 1**]:
   0 = "not at all" to 10 = "as bad as it can be"
   c. **[MOST IMPORTANT IMPROVED SYMPTOM 2**]:
   0 = "not at all" to 10 = "as bad as it can be"
   d. **[MOST IMPORTANT IMPROVED SYMPTOM 3**]:
   0 = "not at all" to 10 = "as bad as it can be"

6. How soon after starting the study medication did you notice an improvement in your symptoms? What, specifically, did you notice first?
   a. [Probe specifically on the timing for each OF THE TOP 3 improvement areas, if patient can recall]

IV. IMPACT OF IMPROVEMENTS

7. You have mentioned some of the most important improvements you have noticed since you started the study medication. Can you describe how you have experienced these changes and the impact they have had on your daily life?
   a. How did improvement with **[IMPROVED SYMPTOM 1]** change or impact your life?

   Probe on reasons for importance (e.g., improvement in…):
   - ☐ mood
   - ☐ family
   - ☐ work or school
   - ☐ social life
     - interacting with others
     - ability to relate to or get along with others (family and friends)

- ☐ sleep (daytime sleepiness or ability to sleep)
- ☐ finances
- ☐ cognition, thinking or memory
- ☐ energy/motivation

[REPEAT 2 MORE TIMES: PROBE ON EACH OF THE TOP 3 MOST IMPORTANT IMPROVED SYMPTOMS]

V. OVERALL EFFICACY AND SATISFACTION

For these next few questions, please think about your depression and related symptoms before you started the study medication compared to how you are feeling now (today). Choose the one response that best describes the change you have experienced.

8. Overall, since you started the study medication would you say that your **[IMPROVED SYMPTOM 1] is**…**[read response options as many times as participant needs to select a response]**

    1 - A great deal better
    2 - Moderately better
    3 - A little better
    4 - No change
    5 - A little worse
    6 - Moderately worse
    7 - A great deal worse

    a. What does **[patient's response to this item]** mean to you?

9. Overall, since you started the study medication would you say that your **[IMPROVED SYMPTOM 2]** is…

    1 - A great deal better
    2 - Moderately better
    3 - A little better
    4 - No change
    5 - A little worse
    6 - Moderately worse
    7 - A great deal worse

    a. What does **[patient's response to this item]** mean to you?

10. Overall, since you started the study medication would you say that your **[IMPROVED SYMPTOM 3]** is…

    1 - A great deal better
    2 - Moderately better

3 - A little better
4 - No change
5 - A little worse
6 - Moderately worse
7 - A great deal worse

a. What does **[patient's response to this item]** mean to you?

11. Overall, how satisfied were you with the study medication's ability to relieve your **[IMPROVED SYMPTOM 1]**?

0=Not Applicable

1=Not at all satisfied

2=A little satisfied

3=Moderately satisfied

4=Quite satisfied

5=Very satisfied

12. Overall, how satisfied were you with the study medication's ability to relieve your **[IMPROVED SYMPTOM 2]**?

0=Not Applicable

1=Not at all satisfied

2=A little satisfied

3=Moderately satisfied

4=Quite satisfied

5=Very satisfied

13. Overall, how satisfied were you with the study medication's ability to relieve your **[IMPROVED SYMPTOM 3]**?

0=Not Applicable

1=Not at all satisfied

2=A little satisfied

3=Moderately satisfied

4=Quite satisfied

5=Very satisfied

Interview date ________________(DDMMMYYYYY)

Study ____ Patient Study ID# __________

VI. SUMMING UP

Thank you for your sharing your thoughts and experiences with us today. This information is very valuable and will help the pharmaceutical company sponsoring this study understand more about depression and related symptoms and to measure improvements in these symptoms. Is there anything else you think we should know (i.e., anything we should have asked but didn't)?

[Thank participant again and end call]

Appendix 2. Illustrative quotations from the exit interviews

Calmness-associated quotations

Agitated (less): I haven't been dealing with anxiety and I'm less agitated.

Aggravated (less): I'm less aggravated, not upset. I'm feeling more patient. Let's put it that way.

Aggression (less): Yeah, I think the aggression has gotten better.

Anger (less): I feel like my ability to control my anger has increased considerably. I don't really feel angry… I don't feel as angry as I used to.

Anxiety (less): I definitely feel less anxious than before.

At ease (more): I think it definitely helped with just feeling more at ease with situations, and just having a better acceptance.

Calm (more): I'm no longer edgy with people and more calm. I guess I can look at the situation more peacefully and look for the positive.

Edginess (less): I feel a little less edgy… and I'm trying to be as polite and courteous to people as I possibly can without pissing them off.

Fearful (less): I am a little less fearful of gloom and doom and bad things happening.

Fidgety (less): I can stand in one place for a long time. I can sit in one place for a long time. I don't have to rearrange myself on a regular basis.

Frustrated (less): I don't get as frustrated as easily.

Hostility (less): I guess the third most important thing or the best improvement I've noted would be, probably, less hostility.

Impulsiveness (less): Well, about the impulsiveness, I've noticed a major improvement on that because I have just stopped completely with the buying things, and I've saved a lot of money. I noticed it, but I only noticed it once I've had all the money saved.

Irritability (less): Definitely, the irritability's the biggest improvement. I have little to none anymore.

Knot in chest/stomach (resolved): Oh, my God, the knot in my stomach is gone.

Mellow (more): Now I'm a little bit more mellowed out because of this.

Nervous (less): I'm not so nervous around other people, and I'm not so nervous being by myself.

Overwhelmed (less): I don't feel as overwhelmed.

Panic/panic attack (less): I was having the panic attacks, probably I would say maybe once a week or once every 2 weeks probably… But now I haven't had one in a long time.

Patient (more): I'm more patient in how I deal with my kids.

Peaceful (more): I'm more at peace.

Physical tightness/stiffness (less): The stiffness [improved].

Relaxed (more): I'm just more relaxed now.

Restlessness (less): I don't feel as restless… because I have a good night's rest.

Shakiness (less): One other thing that's improved is I'm not as shaky. I don't know if that's a result of… I used to have quite a bit of shakes. I don't know what that's about, but I'm not as shaky.

Sit still (improved): I can sit still for a long period of time and just watch TV or just find something in the house to do, just sit down and watch a movie or talk to my daughter or stuff like that. So that got better.

Stress/stressed over things (less): Well, obviously, when you don't worry about things, your stress level goes down… So, it's improved my stress level.

Tension (less): It seems like I had a tension across my abdomen, under my ribs and everything that has loosened up. I know it's not visible. It's just that I had a tension there that is no longer there.

Worry (less): I don't worry so much about little things anymore.

Life engagement-associated quotations

Emotional domain: My mood is much more positive, much better; I don't really feel flat anymore at all, just more positive, I guess… there's more hope there. Well, it's just there's been a big improvement. I feel much better about myself and feel much better about my situation right now.

Physical domain: I have energy to do what I need to do and be excited about it. I just feel more motivated to do stuff instead of just thinking about it and not doing it. I'm actually thinking about it and taking action on it, which is very strange and different. I haven't done that in a long time.

Social domain: I'm walking the dog; I've started a walking group in my apartment complex. Yesterday, I had coffee with a woman that I just recently met. She's my mother's age. Now, I feel like it's easier to interact with people.

Cognitive domain: I wasn't able to concentrate on things; I couldn't keep my mind on things, but my concentration improved; my mind is a lot clearer, not that foggy thinking, muddled thinking.

Multiple domains (physical + social): I have more energy since I started taking the drug: being able to function again and have conversations with people, getting around, getting my house cleaned, get to work and joke around with people at work.

Appendix 3. Additional results

Supplementary Table 1. Patient-reported improvement codes relating to improvements of any nature (n=104)

Code[a]	n	%
Ability to cope (better)	4	3.8
Activities of daily living (easier)	8	7.7
**Aggravated (less)**	**6**	**5.8**
**Aggressive (less)**	**3**	**2.9**
**Agitated (less)**	**16**	**15.4**
**Anger (less)**	**30**	**28.8**
**Anxiety (less)**	**48**	**46.2**
Appetite (improved; less or more)	30	28.8
**At ease (more)**	**3**	**2.9**
Attitude (improved)	6	5.8
Brain fog (less)	3	2.9
**Calm (more)**	**18**	**17.3**
Chest pain/generalized pain (decreased)	5	4.8
Concentration (improved)	31	29.8
Confidence (increased)	4	3.8
Control of self/emotions (more)	2	1.9
Crying (less)	11	10.6
Desire to live (increased)	3	2.9
Dizziness (less)	1	1.0
**Edginess (less)**	**4**	**3.8**
Emotional stability (more)	6	5.8
Energy (more)	72	69.2
Enjoy quietness (increased)	1	1.0
Enjoy things (more)	11	10.6
Express emotions (more)	3	2.9
Falling asleep (improved)	39	37.5
**Fear (less)**	**5**	**4.8**
Feel emotions (more)	4	3.8
**Fidgety (less)**	**3**	**2.9**
Flat (less)	3	2.9
Focus (more)	29	27.9
**Frustration (less)**	**3**	**2.9**
Headaches (gone/improved)	7	6.7
Helplessness (less)	1	1.0
Hopelessness (less)	13	12.5
**Hostility (less)**	**4**	**3.8**
**Impulsivity (less)**	**13**	**12.5**
Interest in things (more)	5	4.8
**Irritability (less)**	**46**	**44.2**
**Knot in chest/stomach (resolved)**	**2**	**1.9**
Libido (improved)	3	2.9
**Mellow (more)**	**2**	**1.9**
Memory (improved)	10	9.6
Mood (improved) – happier/less sad	62	59.6
Motivation (more)	72	69.2

**Code**[a]	**n**	**%**
**Nervousness (less)**	**7**	**6.7**
Numb (less)	2	1.9
Optimistic/positive outlook (more)	30	28.8
**Overwhelmed (less)**	**4**	**3.8**
**Panic/panic attacks (less)**	**5**	**4.8**
**Patient (more)**	**11**	**10.6**
**Peaceful (more)**	**2**	**1.9**
**Physical tightness/stiffness (less)**	**2**	**1.9**
Productive (more)	18	17.3
**Relaxed (more)**	**13**	**12.5**
Rested (more)	7	6.7
**Restlessness (less)**	**4**	**3.8**
Rumination (less)	3	2.9
Self-worth/esteem (improved)	12	11.5
**Shakiness (less)**	**3**	**2.9**
**Sit still (improved)**	**6**	**5.8**
Sleeping less/staying alert (improved)	14	13.5
Social interaction (improved)	66	63.5
Spent time in bed (less)	2	1.9
Staying asleep (improved)	43	41.3
**Stress/stressed over things (less)**	**7**	**6.7**
Suicidal/suicidal thoughts (less)	3	2.9
**Tension (less)**	**9**	**8.7**
Thought process/thought clarity (improved)	16	15.4
Tired (less)	12	11.5
Tolerant (more)	4	3.8
Wellbeing (better)	2	1.9
**Worry (less)**	**18**	**17.3**

[a]Codes relating to calmness are presented in bold.

Supplementary Table 2. Assignment of life engagement codes to exit interview vocabulary (n=105)

Patient #	Study #	Domain Code				Patient Vocabulary
		E	P	S	C	
1	1	1	1	0	0	Less aggravated; not as sad; less overwhelmed; don't feel hopeless; less emotionally tired; calmer; energy/motivation
2	1	0	1	0	0	Motivation; energy; sleep
3	1	1	1	1	1	Highly functional; less irritation; desire to live; hopeful; get more done; relating to people; more involved in life; more outgoing; sleep; excited to start the day
4	1	1	1	1	0	More positive outlook; motivation/energy; sleep; less flat; hopeful
5	1	1	1	1	0	Energy; motivation; out in public more; socialize; less edgy; less fearful; less irritable
6	1	1	1	0	0	More positive perspective; more positive; less angry/snapping/ irritable; sleep; energy/motivation
7	1	1	1	1	0	Motivation; sleep; do more things; less sad
8	1	1	1	1	0	Does more things/social engagement; sleep; motivation
9	1	0	0	0	0	N/A (no improvements)
10	1	0	1	1	0	Sleep; motivation; appetite; less impulsive
11	1	1	1	1	1	Social engagement; energy; attitude/mood improved; uppity; calmer; cheering up; brighter; motivated
12	1	1	1	1	1	More positive outlook; motivation/energy; sleep; socialize more; more confidence
13	1	1	1	1	1	Relaxed/calmer; less impulsive/anger; less panics; energy; laughing/smiling; concentration; positive attitude; assertive; sleep; on the go
14	1	1	1	1	0	Socializing/less isolation; achieving things; sleeping
15	1	1	1	0	1	Motivated; less sad; concentration; better mood; sleep
16	1	1	1	1	0	Social engagement; sleeping; make plans and follow through; motivated
17	1	0	1	0	1	Less irritation/hostility; better short-term memory; energy/ motivation; sleep
18	1	0	1	0	0	Sleep; motivation
19	1	1	0	0	1	Motivation (but not energy); more enjoyment; concentration
20	1	1	1	0	1	Less sad; less irritated/annoyed/frustrated; energy; clear mind/not foggy; less feeling of dread; concentrate; more inspired/upbeat/ motivated
21	1	0	0	0	0	N/A (no improvements)
22	1	0	0	0	0	N/A (no improvements)
23	1	1	1	1	0	Energy/motivation; less irritation; interest/enjoyment in doing things; humor/connect with others
24	1	1	1	1	1	Energy; pay attention; finish tasks; concentration
25	1	1	1	1	1	Improved mood; look forward to future; doing more (cleaning); energy; motivation; sleep; memory; concentration/focus; increased attention span
26	1	1	1	1	1	Energy; concentration; confidence; control; general outlook/mood; not flat anymore/wide range of appropriate emotions; sleep
27	1	0	1	1	0	Motivation; sleep
28	1	0	0	0	0	N/A (no improvements)
29	1	1	1	1	0	Less agitated/irritated; happy; less worry; more productive/getting things done; connect more with others; in control; no racing thoughts
30	1	0	0	0	0	N/A (no improvements)
31	1	1	1	0	0	Slight improvement in motivation; some sleep improvement

Patient #	Study #	Domain Code				Patient Vocabulary
		E	P	S	C	
32	1	1	1	0	1	Energy; concentration; sleep; focus; memory
33	1	1	1	1	0	Energy; motivation; socialize more; get more done; feel better about herself
34	1	1	1	1	1	Sleep; energetic; less impulsive; able to get more things done; less depressed mood
35	1	1	1	0	1	Normal range of emotions; motivated; thought process (not negative all the time); energy level; concentration improved; happier mood
36	1	1	1	1	0	Less agitated/anger/irritation; motivation/energy; gone out more; not as sad; sleep
37	1	1	1	0	0	Better outlook; energy; getting more done; look forward to things
38	1	1	1	1	0	Less agitated/anger; aggression; sleep; energy; motivation/drive; calls friends; knows she has value
39	1	1	1	1	1	Hopeful; motivation/energy; thinking more clearly; more balanced; less fog; focus
40	1	1	1	0	0	Less irritation; sleep; mood; motivation/energy
41	1	0	1	0	1	Motivated; concentration; attention span; sleep
42	1	0	0	0	0	N/A (no improvements)
43	1	1	1	1	0	Feels less flat; socialize; a bit more motivated
44	1	1	0	0	0	Little bit more motivated; less sad; likes being in the quiet and being alone; sleep
45	1	0	0	0	0	N/A (no improvements)
46	1	1	1	1	0	Less aggravated; motivated; less sad; getting out and interacting with people/socializing
47	1	1	0	0	0	Less sad/more positive; less irritation/agitation; more patient
48	1	1	0	1	0	A little better/good outlook; less irritable
49	1	0	0	0	0	N/A (no improvements)
50	1	1	1	0	0	Happiness; sleep; appetite; energy; motivation; upbeat
51	1	1	1	0	1	Better mood; less agitated; sleep; concentration; energy/ motivation; mental clarity
52	2	0	0	0	0	No comments
53	2	1	1	0	0	Bit better motivation; worse concentration
54	2	0	1	1	1	Less anxiety/less edgy around people; less irritation; motivation; energy; mental sharpness
55	2	1	0	1	0	Sleep; happier/optimistic; less anxious; less numb
56	2	1	1	1	1	Less aggravated; happier; wants to do things; energy; focus; motivation; enjoyment
57	2	1	0	0	0	Deals with things better; calm; less irritated
58	2	1	1	1	1	Socialize; less irritable; happier; calmer; focused; not in a fog; goals
59	2	1	1	1	1	Concentration; get things done; energy; interested in doing things; no more hateful thoughts
60	2	1	1	1	0	Less irritation; less impulsive; motivated; energy; sleep; happy
61	2	1	1	0	1	Energy/motivation; less anger; focus/concentrate; productive; mood
62	2	1	1	0	1	Energy/motivation; concentration; less agitated; positive outlook
63	2	1	1	1	0	Motivation; happier mood; confidence; less irritated
64	2	0	1	0	0	Less irritated; energy/active; patience; sleep
65	2	1	1	0	1	Better outlook; energy; motivation; irritability; anger; concentration/focus
66	2	1	1	1	0	No more thoughts of self-harm; even keel; energy/motivation; happier mood; less angry; happier/smile more; sociable

Patient #	Study #	Domain Code				Patient Vocabulary
		E	P	S	C	
67	2	1	1	0	1	Motivation; concentration; energy; less down; sleep; go out; get things done
68	2	1	1	0	0	Energy/motivation; less irritation; less anger
69	2	1	0	0	1	Happier mood; less irritation; less impulsive; concentration/focus
70	2	1	1	0	0	Well-being (don't want to harm himself or others)
71	2	1	1	1	0	Socialize; get things done; energy
72	2	1	1	1	1	Less irritated; motivation; energy; focus/concentration; socializing
73	2	1	1	0	0	Energy/motivation; get things done; sleep
74	2	1	1	0	0	Motivation/energy; get things done; less angry/irritable
75	2	1	1	0	0	Energy; less irritated; more patient; motivation; interest in doing things
76	2	1	1	0	0	Optimistic/hopeful about future; energy; no change in motivation/ interest in doing things
77	2	0	1	0	1	Concentration/memory; less irritation/anger; energy
78	2	1	0	1	0	Less irritation/anger; socialize; happy/joyful
79	2	1	1	1	1	Better mood; peace of mind; sleep; focus
80	2	1	0	0	0	Sleep; mood; calmer/less panicky
81	2	0	0	0	0	N/A (no improvements)
82	3	0	0	0	0	N/A (no improvements)
83	3	1	1	1	0	Sleep; motivation; less irritable
84	3	1	0	1	0	Less tense/anxious; enjoying things more; feels less down; less irritable
85	3	0	0	0	1	Less irritation; less stress; less impulsive; memory; less anger
86	3	0	0	0	0	N/A (no improvements)
87	3	1	1	0	1	Less irritation; concentration; sleep; feeling better about herself and future/positive
88	3	1	0	0	0	Calmed down depression; sleep
89	3	1	1	0	0	Patience; energy/motivation; mood
90	3	1	1	1	1	Less irritability; motivation; energy; lifted spirits; concentration; happier; socialize
91	3	1	1	0	1	Less anger; energy; motivation; focus/concentration; better perspective; sleep; patience
92	3	1	1	0	0	Less anger; sleep; mood; energy; motivated; desire
93	3	1	0	0	0	Happier; motivated; sleep
94	3	1	1	0	1	Less agitated; focus; energy
95	3	1	1	0	0	Motivation; energy; less impulsive
96	3	1	1	1	0	Energy; socialize; happy; patience; calm
97	3	1	1	1	1	Better mood; motivation/energy; get more done; less angry; calm; concentrate
98	3	1	0	0	0	Mood; less anger/irritability
99	3	1	1	0	1	Mentally clear; committed to getting things done; concentrate
100	3	0	1	0	0	Less angry; energy; not self-critical; active
101	3	1	1	0	1	Better mood; motivation/energy; concentration/focus
102	3	1	1	0	0	Hopeful; energy; positive thoughts
103	3	1	1	1	0	Socialize; mood; motivation
104	3	0	1	0	1	Energy; sleep; focus
105	3	1	1	1	0	Socializing; enjoying life; calm; content; less anger; getting things done; sleep

Abbreviations: C=cognitive; E=emotional; N/A=not applicable; P=physical; S=social.
